# Pathophysiological Significance of Store-Operated Calcium Entry in Megakaryocyte Function: Opening New Paths for Understanding the Role of Calcium in Thrombopoiesis

**DOI:** 10.3390/ijms17122055

**Published:** 2016-12-08

**Authors:** Christian A. Di Buduo, Alessandra Balduini, Francesco Moccia

**Affiliations:** 1 Department of Molecular Medicine, University of Pavia, 27100 Pavia, Italy; christian.dibuduo@unipv.it; 2 Laboratory of Biotechnology, Istituto di Ricovero e Cura a Carattere Scientifico (IRCCS) San Matteo Foundation, 27100 Pavia, Italy; 3 Department of Biomedical Engineering, Tufts University, Medford, MA 02155, USA; 4 Laboratory of General Physiology, Department of Biology and Biotechnology “Lazzaro Spallanzani”, University of Pavia, 27100 Pavia, Italy

**Keywords:** megakaryocyte, platelet production, calcium, SOCE, calreticulin, myelofibrosis

## Abstract

Store-Operated Calcium Entry (SOCE) is a universal calcium (Ca^2+^) influx mechanism expressed by several different cell types. It is now known that Stromal Interaction Molecule (STIM), the Ca^2+^ sensor of the intracellular compartments, together with Orai and Transient Receptor Potential Canonical (TRPC), the subunits of Ca^2+^ permeable channels on the plasma membrane, cooperate in regulating multiple cellular functions as diverse as proliferation, differentiation, migration, gene expression, and many others, depending on the cell type. In particular, a growing body of evidences suggests that a tight control of SOCE expression and function is achieved by megakaryocytes along their route from hematopoietic stem cells to platelet production. This review attempts to provide an overview about the SOCE dynamics in megakaryocyte development, with a focus on most recent findings related to its involvement in physiological and pathological thrombopoiesis.

## 1. Introduction

The calcium ion (Ca^2+^) is an ubiquitous signaling entity which plays key role in regulating the functions of virtually all cell types, including proliferation, differentiation, exocytosis, gene transcription, migration and apoptosis [1,2]. The intracellular responses are regulated by a tight control of intracellular cytoplasmic Ca^2+^ concentration ([Ca^2+^]_i_) by the finely tuned interplay of several Ca^2+^-transporting proteins, such as Ca^2+^ pumps, Ca^2+^ binding proteins and Ca^2+^ permeable channels. In resting states, the sarco/endoplasmic reticulum (SR/ER) Ca^2+^-ATPase (SERCA) pump, along with plasma membrane Ca^2+^-ATPase (PMCA) and the Na^+^/Ca^2+^ exchanger (NCX), determine the basal [Ca^2+^]_i_; while, upon stimulation by extracellular signals, [Ca^2+^]_i_ increases by Ca^2+^ release from intracellular stores or extracellular Ca^2+^ influx into the cell [3].

The main intracellular Ca^2+^ store is represented by the SR/ER. However, using genetically targeted Ca^2+^ reporter proteins, like aequorin and the cameleons, together with detailed immunocytochemical mapping and functional assays, contributions from additional organelles such as the Golgi apparatus, the lysosomes, the mitochondria, the nuclear envelope, and the secretory granules have been identified [1,4,5,6].

Ca^2+^ release from intracellular stores is usually determined by the activation of the phospholipase C (PLC) pathway by G-Protein Coupled Receptors (GPCRs) or Tyrosine Kinase Receptors (TKRs) [7]. PLC cleaves phosphatidylinositol 4,5-bisphosphate (PIP2) into the second messengers inositol 1,4,5-trisphosphate (IP3) and diacylglycerol (DAG). DAG engages both protein kinase C and non-selective cation channels on the cellular membrane, such as Transient Receptor Potential Canonical (TRPC) 3, TRPC6 and TRPC7, whereas IP3 rapidly diffuses within the cytosol to bind to and open ER-embedded IP3 receptors (IP3Rs). IP3Rs, in turn, serve as Ca^2+^-permeable channels to release luminal stored Ca^2+^. In addition to IP3, cyclic adenosine diphosphate (ADP) ribose triggers Ca^2+^ release from the ER by gating ryanodine receptors (RyRs) [8], while nicotinic acid adenine dinucleotide phosphate mobilizes Ca^2+^ stored within the acidic Ca^2+^ stores of the endolysosomal system [9].

Extracellular Ca^2+^ influx can occur through various pathways. A variety of different Ca^2+^-permeable channels have been found to coexist in the plasma membrane including voltage-operated channels (VOCs), second messenger-operated channels (SMOCs), receptor-operated channels (ROCs) and store-operated channels (SOCs) [3]. VOCs are activated by membrane depolarization and are found in excitable cells, like nerve and muscle cells, but are largely excluded from nonexcitable cells. SMOCs, found in some excitable and nonexcitable cells, are activated by small messenger molecules, the most common being IP3, cyclic nucleotides, and lipid-derived messengers (DAG, arachidonic acid and its metabolites). ROCs, preponderant in excitable cells, open rapidly upon binding an external ligand that is usually a neurotransmitter or a hormone. Finally, SOCs are activated by the depletion of intracellular Ca^2+^ stores, according to a mechanism termed Store-Operated Ca^2+^ Entry (SOCE). SOCs appear to be widespread in nonexcitable cells, although they are also present in excitable cells [10,11,12], existing in all eukaryotes from yeast [13] to humans [14], thus probably representing the primordial and best preserved Ca^2+^ entry pathway.

Although originally identified as a mechanism for ensuring the refilling of intracellular stores following Ca^2+^ release [15,16], SOCE is now directly linked to the activation of specific cellular functions, differently regulated depending on the stimulus and the cell type [10], including immune system activation [17], fluid secretion in salivary gland acinar cells [18], neurogenesis and neuronal excitability [12,19], cancer cell migration and metastasis [20], endothelial cell proliferation [21], skeletal muscle contractility [22], smooth muscle migration and proliferation [23,24], cardiac hypertrophy [25], cell cycle and cell proliferation [26], and gene expression [27]. Further, increasing evidences have recently identified the contribution of SOCE in thrombopoiesis, the process that ensures the differentiation of megakaryocytes, the platelet precursors, and final platelet production [28].

Here we will review the mechanisms of SOCE and discuss the most recent findings regarding its involvement in regulating different megakaryocyte functions, from proliferation to platelet formation. Finally, we will provide evidence that an alteration of SOCE activity may concur to the development of human pathologies.

## 2. Dissecting the Molecular Mechanisms of Store-Operated Calcium Entry

The physiological hallmark of SOCE is the long-lasting plateau phase that follows the initial IP3-dependent intracellular Ca^2+^ release induced by extracellular stimulation. Specifically, the first signal is generated upon binding of cytokines, growth factors, hormones, and neurotransmitters to their specific receptors, which leads to the generation of the second messenger IP3 and consequent mobilization of the IP3-sensitive Ca^2+^ pool. In the second phase, the decrease in ER Ca^2+^ content causes the activation of plasma membrane Ca^2+^ channels resulting in the influx of extracellular Ca^2+^ inside the cells [10,29]. This mechanism was first described by Putney in 1986 as “capacitative Ca^2+^ entry”, when he proposed that the amount of Ca^2+^ in the stores of acinar parathyroid cells controls the extent of Ca^2+^ influx and store refilling and compared it to the arrangement of resistor (channel) and capacitator (Ca^2+^ store) in an electrical circuitry [15]. Nearly after, the evidence of the existence of a store-operated Ca^2+^-selective membrane current with a largely positive reversal potential (*E*_rev_ = +60/+70 mV), that arose in response to different Ca^2+^ store depletion strategies, further supported the hypothesis that highly selective Ca^2+^-Release Activated Ca^2+^ (CRAC) channels could be activated in response to ER Ca^2+^ emptying [30,31]. The molecular structure of CRAC channels has been fully dissected in the last decade by carrying out an extensive function-based genetic screen by systematic Ribonucleic Acid (RNA) interference conducted on a subset of candidate genes in *Drosophila* S2 cells and HeLa cells [32,33,34,35]. This approach led to the identification and characterization of the Stromal Interaction Molecule (STIM) family, which represents the ER Ca^2+^ sensor, and of the Orai family, which provides the pore-forming subunit of CRAC channels, that cooperate in an elegant signaling mechanism to ensure SOCE activation.

### 2.1. Stromal Interaction Molecules: The Calcium Sensor of Intracellular Stores

STIMs, first identified in 2005, function as Ca^2+^ sensors in the ER and control CRAC channel opening in both quiescent and stimulated cells [32,33,36,37]. This family encompasses two members, STIM1 and STIM2; however, most studies have concluded that the ER-localized STIM1 is the main isoform involved in SOCE activation upon extracellular stimulation [32,38,39], while STIM2 controls Ca^2+^ entry in resting cells [40].

STIM1 is a single-pass transmembrane (TM) protein of 665 amino acids and ≈77 kDa that is abundant in the ER membrane [32,39], but can be also expressed on the plasma membrane, although at a minor extent [36,41]. When embedded within the ER membrane, STIM1 is oriented such that the N-terminus appears within the lumen and the C-terminus in the cytoplasm. The protein is comprised of several identifiable structural and functional motifs that are shared with STIM2 [10]. The luminal side contains a Ca^2+^ binding canonical EF-hand domain (cEF), which confers the protein with sensitivity to ER Ca^2+^ levels, a hidden non-canonical EF-hand domain (hEF) that does not bind Ca^2+^, and a sterile-α motif (SAM), that is required for protein-protein interaction during the oligomerization process (see below) [10,32,33]. SAM is followed by a TM domain which is followed by three conserved CC domains (CC1, CC2 and CC3) and polybasic lysine-rich (K) domain at the very end of the C-terminus which mediate, respectively, STIM1 binding to Orai1 and membrane phospholipids. More specifically, Orai1 is recruited and gated by the CRAC activation domain (CAD; also termed STIM-Orai-activating region or SOAR or coiled-coil domain b9 or CCb9) of STIM1, which encompasses CC2 and CC3, while the K-domain anchors STIM1 to the inner leaflet of the plasma membrane [10,32,42,43]. STIM2, the second member of the vertebrate STIM protein family, is exclusively present in the ER membrane [44], shows 61% amino acid homology and similar domain architecture to STIM1 [45], but presents a lower Ca^2+^ binding affinity [46]. The variety of STIM proteins is further enhanced by the existence of diverse splice variants, namely STIM1L [47] and three STIM2 splice variants, STIM2.1 (also known as STIM2β), STIM2.2 (or STIM2α) and STIM2.3 [48,49]. Of these, STIM2.1 is a positive regulator of Orai1, while STIM2.2 inhibits CRAC currents and the function of STIM2.3 is still unknown.

### 2.2. The Interplay between Stromal Interaction Molecule and Orai Activates the Complex Choreography of Store-Operated Calcium Entry

In mammals there are three Orai genes that encode for Orai1, 2 and 3 proteins which function as pore forming subunits of CRAC channels in different cellular contexts [10,12,20,42,50]. The name Orai was given on the basis of Greek mythology (Orai are the keepers of heaven’s gate) after they were established as the long sought mediator of CRAC currents in immune cells [51]. Each Orai channel consists in a ≈30 kDa monomer that comprises four TM domains flanked by cytosolic N- and C-termini and linked by one intracellular and two extracellular loops. The TM segments of each Orai isoform share 81%–87% pairwise sequence identity [52]. However, only Orai1 has an N-terminal proline- and arginine-rich region that could take part to channel gating [52]. The C-terminus of each Orai isoform contains a CC domain which is also required for the physical interaction with STIM1 [53,54]. Although it has long been thought that Orai subunits are assembled into a tetrameric channel [10], the crystal structure of *Drosophila* Orai (dOrai) unveiled a hexameric organization [55]. The six subunits are arranged around a central pore, which is lined exclusively by TM1 and by an α-elical extension of the N-terminal TM1-proximal segment (residues 74–90, ETON region) [55,56]. Importantly, the ETON region provides a further binding interface for STIM1 during the gating process [56]. A ring of glutamate residues (E106) at the outer mouth of the channel pore constitutes the Ca^2+^ selectivity filter, towards which extracellular Ca^2+^ is attracted by three negatively charged aminoacids (D110, D112, and D114) located within the external vestibule. The selectivity filter is followed by a hydrophobic environment with three turns of hydrophobic residues (V102, F99, and L95), of which V102 serves as activation gate [57] and represents a barrier to Ca^2+^ flux in the closed Orai channel [58]. Finally, the lower third region is lined by three α-elical basic aminoacids (R91, K87, R83) which coordinate anions, a rather unusual feature for a cation-selective channel [55]. The channel pore is flanked by three concentric rings subsequently contributed by TM2 to TM4, whereby TM2 and TM3 form the intermediate layers shielding TM1 from the surrounding lipid environment and TM4 constitutes the outermost segment, the one which is presumably most exposed to membrane lipids [55]. The molecular architecture of dOrai needs to be confirmed in humans, but it provides a solid framework to decipher the complex interaction between STIM and Orai proteins.

When Ca^2+^ stores are full and the ER Ca^2+^ concentration ranges between 400 and 600 µM, STIM1 forms dimers which are homogeneously distributed throughout the ER membrane due to their ability to rapidly diffuse along the microtubules [10]. Upon IP3-dependent Ca^2+^ mobilization, Ca^2+^ dissociates from the cEF hand, thereby triggering a complex sequence of intra- and intermolecular interactions that culminate in STIM1 activation, multimerization, and relocation into defined ER-plasma membrane junctions. Herein, STIM1 may finally bind to and gate Orai1, thereby activating the Calcium Release-Activated Calcium Current (I_CRAC_) [10,42,59]. Each Orai isoform carries a Ca^2+^-selective current with well-defined biophysical and pharmacological features. The fingerprint of the I_CRAC_, which represents the archetypal store-operated current, has been defined as follows [10,29,60]: Lack of voltage-dependent activation, prominent inward rectification at negative potentials, reversal potential (*E*_rev_) > +60 mV, Ca^2+^:Na^+^ permeability ratio of 1000:1, unitary Ca^2+^ conductance of 10–35 fS, fast Ca^2+^-dependent inactivation (CDI), high sensitivity to trivalent cation block and biphasic sensitivity to 2-aminoethoxydiphenyl borate (2-APB; activates at <10 µM, but inhibits at 50–100 µM). Orai1 mediates the I_CRAC_ in a growing number of cell types, including, but not limited to, immune cells [61], endothelial cells [62], vascular smooth muscle cells [63], melanocytes [64], microglia [65] and hepatocytes [66].

Although very similar in structure, STIM1 and STIM2 differ in their sensitivity to ER Ca^2+^ levels, their Ca^2+^ dissociation constants (*K*_d_) being, respectively, 200 and 400 µM [40]. As the ER Ca^2+^ concentration ([Ca^2+^]_ER_) ranges between 400 and 600 µM, STIM2 is activated by significantly smaller depletion of the luminal Ca^2+^ pool as compared to STIM1 [40]; moreover, the EF-SAM domains of STIM2 undergoes slower unfolding and self-association kinetics upon Ca^2+^ withdrawal, which reflects in slower and less efficient Orai1 activation [46,67]. It turns out that, while STIM1 drives SOCE upon massive emptying of the ER Ca^2+^ pool, STIM2 controls basal Ca^2+^ entry and sustains the physiological Ca^2+^ oscillations arising in response to moderate-to-weak stimulation [40,68]. The stoichiometry of STIM1-Orai1 coupling is still matter of debate as the I_CRAC_ is exquisitely sensitive to the number of STIM1 subunits interacting with the Orai1 hexamer [69,70]. Several studies reached the conclusion that maximal I_CRAC_ activation occurs at a ratio of 2:1 of STIM1 and Orai1, respectively, while others argued in favor of a 1:1 stoiochiometric ratio [69,71].

## 3. Transient Receptor Potential Canonical Channels: Additional Components of Store-Operated Calcium Entry

The I_CRAC_ is not the sole Ca^2+^-permeable current activated by IP3-dependent depletion of the ER Ca^2+^ store. Electrophysiological recordings carried out on many different cell types revealed the existence of a store-operated current, termed Store-Operated Activated Calcium Current (I_SOC_), which exhibited slightly biophysical properties as respect to the I_CRAC_ [29]. The I_SOC_ is generally permeable to Ca^2+^, Na^+^, K^+^ and Cs^+^ and exhibits a significantly greater conductance than Orai1 channels, ranging from 1 to 43 pS [29]. The I_SOC_ is mediated by members of the TRPC sub-family of non-selective cation channels, which are activated as a consequence of PLC stimulation [72]. TRPC channels are subdivided into four subsets based on their sequence homology: TRPC1, TRPC2, which is a pseudogene in humans, TRPC4/5, and TRPC3/6/7 [73]. Typically, TRPC channels are present in the plasma membrane or in specialized lipid microdomains containing caveolae [74,75]. All members of the TRPC family share a common topology [76]. The cytoplasmic N- and C-termini are separated by six transmembrane domains (TM1-TM6), including a putative pore region (LFW pore motif) between TM5 and TM6 [77,78]. The N-terminus is composed of three to four ankyrin repeats, a predicted coiled-coil region, a putative caveolin binding region and a Protein Kinase G (PKG) phosphorylation sites. The ankyrin repeats and the caveolin binding region appear to be required for correct targeting of TRPC to the plasma membrane [79], while the coiled-coil motif plays a role in the control of TRPC oligomerisation [80]. The cytoplasmic C-terminus includes a Protein Kinase C (PKC) phosphorylation sites, the TRP signature motif (EWKFAR), a highly conserved proline-rich motif, a predicted coiled-coil region and the CIRB (calmodulin/IP3 receptor binding) region which may contribute to plasma membrane targeting [76,81,82,83,84,85]. TRPC channels may assemble in both homomeric and heteromeric complexes, thereby giving rise to a bewildering variety of cationic channels whose biophysical features and physiological roles are yet to be fully dissected. For instance, TRPC1 may associate with TRPC4 and TRPC5, while TRPC3 has the potential to interact with TRPC3 and TRPC7 [77,78,86]. Furthermore, they can form heterometic channel complexes with other members of the TRP super-family. For instance, TRPC1 has been shown to associate with either Transient Receptor Potential Vanilloid 4 (TRPV4) [87], TRPV6 [88], or the Transient Receptor Potential Ankyrin-1 (TRPA1) [89]. As regard to SOCE, all TRPC channels have been associated to the I_SOC_ developing in response to ER Ca^2+^ depletion [90,91]. As discussed elsewhere [90,91], however, the strongest evidence in favor of TRPC channel contribution to SOCE has been provided for TRPC1 [92,93,94] and TRPC4 [95,96,97], while the operation-mode of TRPC3 depends on its expression levels in naïve tissues [98] and TRPCs 5, 6, and 7 serve as receptor-operated channels [99]. The store-sensitivity of TRPC1 and TRPC4 depends on their ability to bind to STIM1. Earlier studies showed that gating of TRPC1 can be gated by the electrostatic interaction between the negatively charged aspartate residues in TRPC1 (^639^DD^640^) with the positively charged lysines in the STIM1 K-domain (^684^KK^685^) [100]. These acidic residues are conserved among all TRPC channels and underlies also STIM1-dependent TRPC4 activation following InsP_3_-dependent Ca^2+^ release [101]. The exact mechanism that determines which TRPC isoforms are recruited by STIM1 upon depletion of the ER Ca^2+^ pool remains elusive, but could involve either their localization at precise sites within the plasma membrane, such as caveolae, or their propensity to interact with Orai1 [90,99]. Intriguingly, several reports revealed that knocking down Orai1 suppressed SOCE despite the presence of endogenous or heterologously expressed STIM1 and TRPC1. STIM1, Orai1 and TRPC1 were found to associate into a heteromeric supermolecular complex in response to ER Ca^2+^ depletion in many different cell types, including in human salivary gland (HSG) cell [102], human platelets [103], human liver cells [104], mouse pulmonary arterial smooth muscle cells [105], human parathyroid cells [106], and rat kidney fibroblasts [107]. Several models have been proposed to interpret this interaction [90,99,108,109]. Orai1 and TRPC1 could both contribute to line the channel pore, each being activated by STIM1 upon store depletion. Alternatively, Orai1 has been suggested to mediate the STIM1-dependent activation of TRPC1, which, in this scenario, would contribute the pore-forming subunit of the store-operated channel [103]. More recently, Ambudkar’s group showed that Orai1 and TRPC1 form distinct STIM1-regulated Ca^2+^-permeable channels. Studies conducted on HSG cells unveiled that Orai1-dependent Ca^2+^ entry results in TRPC1 recruitment to the plasma membrane in close proximity to Orai1; herein, TRPC1 is subsequently gated by STIM1 [110]. As a consequence, the I_SOC_ recorded in these cells is a mixed current composed by the TRPC1/STIM1-mediated non-selective cation current flowing through TRPC1 and by the Orai1/STIM1-mediated I_CRAC_ [90,110]. This mechanism would also explain why STIM1, Orai1 and TRPC1 co-immunoprecipitate upon ER Ca^2+^ depletion in the cellular models described above.

## 4. Thrombopoiesis: The Long Route of Megakaryocytes to Platelet Production

In adult mammals, hematopoiesis occurs in the bone marrow, which supports simultaneously the life-long maintenance of hematopoietic stem cells (HSCs) and the regulated production of end-stage lymphoid, myeloid and erythroid cells [111]. Thrombopoiesis is defined as the process by which mature megakaryocytes are derived from HSCs to produce platelets [112,113], the smallest cells in the human blood (≈3 μm), which perform crucial roles in hemostasis, but also in several other processes such as angiogenesis, immunity, tissue regeneration and wound healing [114].

The first step of megakaryocyte development is regulated by the lineage-specific growth factor Thrombopoietin (TPO) [115] and consists in HSC commitment with arrest of proliferation and initiation of endomitosis, the process by which megakaryocytes increase their nuclear content developing polyploid multilobed nuclei [116,117]. The second step is associated with cytoplasm expansion and intense synthesis of proteins to be delivered into the secretory granules. The most abundant being α-granules, containing Platelet Factor 4 (PF4), von Willebrand Factor (vWF), fibronectin, Platelet Derived Growth Factor (PDGF), Vascular Endothelial Growth Factor (VEGF) and Transforming Growth Factor-β1 (TGF-β1), and δ-granules, enriched with small molecules such as serotonin, epinephrine, adenosine triphosphate (ATP), ADP and ions [118,119,120]. Once mature, megakaryocytes come in close contact with bone marrow sinusoids, under Stromal Derived Factor-1α (SDF-1α) chemo-attraction, and they undergo characteristic changes in cytoskeleton structure with the extension of multiple long pseudopods, called proplatelets, that assemble nascent platelet at their terminal ends [117,121,122]. Finally, proplatelets extend, through the vascular endothelium, into the lumen of sinusoidal vessels, where the release of mature platelets can be attributed to blood hydrodynamics which allow their shedding form the proplatelet shaft, as demonstrated in vivo in mice by multiphoton intravital microscopy [123,124], and confirmed ex vivo in humans, by employing a variety of cell culture techniques and biomimetic platforms reproducing human platelet release [125,126,127,128]. During its lifespan, a mature megakaryocyte can produce up to 10^4^ platelets and each day a human adult produces 10^11^ platelets, a number that can increase in response to acute platelet demand by rapid fragmentation of megakaryocyte cytoplasm [129,130].

The production of platelets is a complex process that involves the support of both bone marrow extracellular matrix (ECM) components and soluble factors. ECMs represent the main bone marrow scaffolding, which surrounds islets of HSCs and committed hematopoietic progenitors [131,132,133]. Within this microenvironment, while differentiating, megakaryocytes may encounter ECMs that differently regulate thrombopoiesis. For instance, it has been described that type I collagen prevents premature platelet release, while supporting megakaryocyte motility [134,135]; while fibronectin and type IV collagen sustain proplatelet formation, but also contribute to the regulation of cell proliferation and differentiation [136,137]. It is known that ECM components are produced by bone marrow stromal cells [138,139,140], however we recently demonstrated that both mouse and human megakaryocytes can actively synthesize and deposit collagens and fibronectin [137,141]. In particular, we showed that TPO is a pivotal regulator of this function by inducing TGF-β1 release, thus controlling ECM component synthesis in an autocrine manner. TGF-β1, as well as other soluble factors (e.g., ADP, VEGF, PF4) and ECM components (e.g., vWF, fibronectin), have been shown to be constitutively released by megakaryocytes to regulate their own differentiation and proplatelet formation [134,136,142,143,144,145,146], indicating that in physiological conditions megakaryocytes can activate an autocrine/paracrine loop which contribute to both their own development and overall bone marrow homeostasis [147]. Consistently, impaired synthesis and release of these proteins has been linked to altered megakaryocyte maturation, proplatelet formation and/or ECMs production [141,142,143,148], leading to a broad spectrum of clinical outcomes, from defective peripheral blood platelet count to deregulated bone marrow homeostasis [147,149,150].

## 5. Biogenesis of Store-Operated Calcium Entry during Thrombopoiesis: Biological Significance in Physiology and Pathology

Recent progresses revealed that most of the signals that regulate platelet production converge into the regulation of the expression and/or activation of SOCE, thus suggesting that Ca^2+^ may have the ability to decode the massages from multiple complex and dynamic inputs ad covert them into a single response having, as major effect, the control of the ordinary course of platelet production. As a consequence, as we will now discuss, impairment of this function may results in pathological phenotypes.

### 5.1. Development of Endoplasmic Reticulum and Endoplasmic Reticulum-Related Proteins in Megakaryocytes

During commitment megakaryocytes can be assigned to distinct stages of maturity according to standard morphological criteria [151]. Specifically, in the early maturation stage, megakaryocytes usually present the lowest cytoplasmic/nuclear ratio, compact nucleus and small size, with fewer and undeveloped non-specific membranous organelles, such as mitochondria, Golgi apparatus, and smooth and rough ER. In the successive stages a progressive cytoplasmic mass increase and appearance of highly lobulated nuclei are accompanied by the expansion of all organelles, especially of Golgi apparatus and ER, which contribute to a continuous membrane supply for the growth of the demarcation membrane system (DMS), an extensive system that provide a membrane reservoir for the formation of future platelets [152,153]. The ER plays primarily a key role in regulating Ca^2+^ signaling through SERCA and IP3Rs. Interestingly, Lacabaratz-Porret et al. analyzed the ER-protein patterns during thrombopoiesis and demonstrated that TPO stimulates the expression and synthesis of SERCA3 throughout megakaryocyte maturation, while SERCA2b is constitutively expressed by megakaryocytes [154]. Importantly, a specific increase in the expression of SERCA3a has been confirmed also in in vitro differentiated human megakaryocytes during proplatelet formation [155]. Further, the presence of IP3R types I, II and III have been shown in the megakaryocytic cell, with the expression of IP3R II and III being slightly up-regulated upon differentiating stimulus [154], consistent with previous findings demonstrating that the expression profile of IP3R subtypes is dynamically modified during hematopoiesis depending on the stimuli that induce differentiation [156]. Therefore, a profound reorganization of the ER and of ER-proteins, involved in promoting store emptying, is achieved during late stages of thrombopoiesis.

### 5.2. Expression and Function of Transient Receptor Potential Canonical during Megakaryocyte Differentiation

Since STIMs and Orais have been discovered and characterized later than TRPCs, early evidence that cells belonging from the megakaryocytic lineage could express genes of the SOCE family was first given in 1997 by Berg et al. [157]. At that time, three human TRPC genes, *TRPC1*, *TRPC2* and *TRPC3*, were shown to be present in different human megakaryocyte cell lines (MEG01, DAMI and HEL), thus suggesting the involvement of TRPCs in regulating Ca^2+^ homeostasis in these cells [157]. These data were confirmed and extended by Wakabayashi et al. who observed SOCE activation in megakaryocytic cell lines, utilizing thapsigargin as a tool to accomplish store depletion, and hypothesized a role for TRPC4 as the molecular component that determines the sensitivity of store-operated channels to intracellular alkalosis in this lineage [158]. The first evidence that primary megakaryocytes express functional SOCE was given by den Dekker et al., who demonstrated the presence of unspliced isoforms for TRPC1, 4 and 6 in human immature (Cluster of differentiation (CD)61/CD42b^low^) and mature (CD61/CD42b^high^) megakaryocytes, differentiated in vitro from human cord blood derived CD34^+^ HSCs [159]. Interestingly, the same group observed high Ca^2+^ influx in megakaryocytes, either upon store depletion by thapsigargin or by the receptor agonist thrombin, in both immature and mature megakaryocytes, thus demonstrating functional activity of SOCE early during the lineage specification [160]. Moreover, they observed an increase in G_q_α and G_i_α1/2 expression in maturing human megakaryocytes, which was accompanied by an increase in intracellular Ca^2+^ signals triggered not only by physiological agonists, such as thrombin and ADP, but also by TPO [160]. Of note, TPO-induced Ca^2+^ signal changed from a single peak in immature megakaryocytes into a series of oscillatory Ca^2+^ spikes in mature cells. It is known that the function of the oscillatory discharges of Ca^2+^ is to produce a sufficiently ample drop in ER Ca^2+^ levels to activate SOCE, which provides the necessary Ca^2+^ influx capable of providing localized signals that specifically couple to downstream effector pathways regulating cellular functions [161,162,163]. For instance, Ca^2+^ oscillations could confer cell survival and drive differentiation in different cell types [164,165,166,167]. Therefore, TPO-induced Ca^2+^ signaling may be involved in modulation of megakaryopoiesis. To this regard, more recently, Ramanathan and Mannhalter showed that TRPC6 increases form early stages of megakaryocytic commitment to mature megakaryocytes to regulate TPO-induced cell proliferation through a store-independent Ca^2+^ entry pathway [168], indicating that a combined effort of SOCE and non-SOCE TRPC channels is involved in regulating Ca^2+^ flows in megakaryocytes. The expression of TRPC1 and TRPC6 was also confirmed in murine megakaryocytes [169]. Specifically, the individual selection of these cells directly from bone marrow specimens allowed the demonstration of a major role for TRPC6 in allowing Ca^2+^ influx upon physiological stimulation with ADP. However, TRPC1^−/−^ and TRPC6^−/−^ mice did not show defective thrombopoiesis as indicated by normal platelet count and size in the peripheral blood [170,171]. Noteworthy, the evidence that megakaryocytes express different isoforms of other store-operated channels, make it difficult to believe that a single knockdown may significantly affect megakaryocyte function.

### 5.3. NF-κB Pathway Is a Major Regulator of Orai Expression in Megakaryocytes

An important next-step toward a better comprehension of the role of SOCE in megakaryocyte physiology was made after the identification of STIM and Orai families as major determinant of SOCE. Since that, Orai1 expression was observed in the human megakaryocytic cell line MEG-01 [172,173]. Borst et al. demonstrated that Orai1 expression is modulated by the serum- and glucocorticoid-inducible kinase 1 (SGK1) [172], a kinase belonging to the AGC family of serine/threonine protein kinases [174,175], that can be regulated by a variety of different triggers, including hormones, thrombin, oxidative stress and growth factors [175]. Specifically, they showed that SGK1 regulates Orai1 expression in megakaryocytes through a nuclear factor κ-light-chain-enhancer of activated B cells (NF-κB) dependent pathway which in turn ensures physiological thrombopoiesis, whereas platelets form *sgk1*^−/−^ mice displayed a significantly blunted SOCE and agonist-induced increased [Ca^2+^]_i_ resulting in impaired platelet activation [172]. Further evidence about the relevance of this pathway was provided by the observation that megakaryocytic cell transfection with the NF-κB p50/p65 heterodimer significantly increases STIM1/Orai1 transcription and protein levels, while 1,25(OH)_2_ vitamin D_3_ decreases STIM1/Orai1 expression and I_CRAC_ in megakaryocyte by negatively modulating NF-κB activity [176]. Moreover, TGF-β1, that is increasingly released during megakaryocyte differentiation regulating platelet production [142], has been recently shown to be a stimulator of SOCE in megakaryocytes, via the up-regulation of SGK1, which in turn activates nuclear factor NF-κB and stimulates Orai1 expression [177]. Interestingly, Almilaji et al. demonstrated that TGF-β1 significantly up-regulates also Na^+^/Ca^2+^-exchanger activity in murine bone marrow megakaryocytes, thus influencing megakaryocytic Ca^2+^ signaling not only by augmenting Ca^2+^ entry, but also by stimulating Ca^2+^ extrusion [178].

### 5.4. Store-Operated Calcium Entry Finely Regulates Physiological Megakaryocyte Functions

The activity of the molecular effectors of SOCE has been shown to be finely regulated during megakaryocyte differentiation. Particularly, Albarrán et al. demonstrated that the TRPA1, a negative regulator of STIM1 and Orai1 interaction, is down-regulated in late phases of megakaryocyte differentiation in order to confer enhanced SOCE functionality to mature megakaryocytes and released platelet particles [89]. The relevance of this finding was clarified in 2014 when our group investigated for the first time the mechanistic link between Ca^2+^ signaling and thrombopoiesis [28]. Specifically, we showed that in vitro differentiated megakaryocytes, from human cord blood derived CD34^+^ HSCs, express all the molecular candidates to mediate SOCE, including STIM1, Orai1, and TRPC1, and that pharmacological-induced intracellular Ca^2+^ release from ER by cyclopiazonic acid promotes their active interaction and consequent extracellular Ca^2+^ flow inside cell cytoplasm [28]. In this context, different functional and biochemical assays evidenced a compartmentalized distinct role of the two Ca^2+^ release/entry routes in response to ADP [28], an autocrine modulator of proplatelet formation [143,179]. Specifically, IP3-dependent Ca^2+^ mobilization from intracellular stores was primarily involved in the activation of biochemical signaling cascades (e.g., Akt, Erk1/2) that promote proplatelet formation, while extracellular Ca^2+^ entry was mainly responsible for the regulation of contractile forces that favors megakaryocyte sensing of extracellular substrates and motility in adhesion to different ECM components (e.g., fibronectin and type I collagen) [28]. In the light of the previous demonstration that human mature megakaryocytes display a significant increase in [Ca^2+^]_i_ upon collagen stimulation [180], these findings support the involvement of SOCE in regulating megakaryocyte interaction with the bone marrow microenvironment, that in turn support proliferation and platelet production [137]. Interestingly, Ca^2+^ entry in megakaryocytes in response to ADP can be amplified by glutamate-induced activation of ionotropic *N*-methyl-d-aspartate receptors (NMDARs), resulting in increased cell proliferation, rather than differentiation, while NMDAR antagonists reduce cell growth and promote differentiation of leukemic megakaryoblasts [181].

All together, these data suggest that a tight control of [Ca^2+^]_i_ is achieved by megakaryocytes along their route to platelet production by different stimuli that integrate in order to ensure the fine balance between cell proliferation and differentiation.

### 5.5. Over-Activated Store-Operated Calcium Entry Is Observed in Pathological Thrombopoiesis

A pronounced megakaryocyte hyperplasia has been shown in heterozygous mice expressing an activating EF hand mutant of STIM1 (*STIM1*^Sax/+^), resulting in the constitutive activation of SOCE and consequent high cytoplasmic Ca^2+^ levels [182]. Macrothrombocytopenia and an associated bleeding disorder were also observed in these mice, due to high basal intracellular Ca^2+^ levels in circulating platelets responsible for a pre-activation state and consequent increased platelet consumption [182], further supporting the evidence that a fine regulation of SOCE in maturing megakaryocyte is crucial to ensure physiological thrombopoiesis. Of note, the megakaryocytic hyperplasia in *STIM1*^Sax/+^ mice was accompanied, at the age of 6 months, by the appearance of bone marrow fibrosis and severe splenomegaly [182], all symptoms resembling characteristic features of Primary Myelofibrosis (PMF), an hematopoietic malignancy belonging to the family of Phildelphia-negative Myeloproliferative Neoplasms (MPNs) [183,184]. It is known that patients affected by PMF present increased deposition of ECM components within bone marrow, resulting in a progressive fibrosis that alters the bone marrow microenvironment architecture, compromising efficient platelet production and overall hematopoiesis, which moves into the spleen leading to splenomegaly, further worsening the prognosis of affected patients [185]. Interestingly, bone marrow megakaryocytes are considered key players in the PMF pathogenesis because of the alteration of both their number and morphology (hyperplasia and dysplasia) [142,186,187]. Further, proplatelet formation in PMF has been described to present several structural alterations, and platelet count may vary from low to abnormally high numbers, with thrombosis as main additional cause of reduced patient survival [186,188]. Importantly, progression to myelofibrosis (secondary myelofibrosis) may occur also in patients affected by Essential Thrombocythemia (ET), another MPN characterized by megakaryocytic hyperplasia and elevated platelet count.

**Figure 1:**
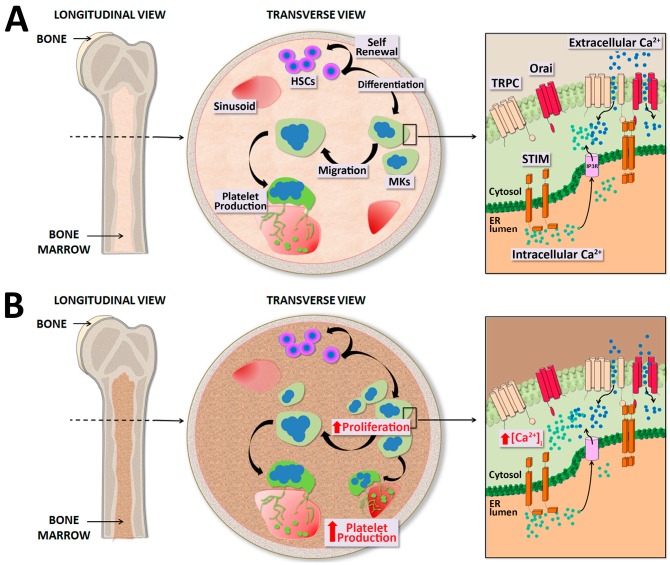
Store-Operated Ca^2+^ Entry (SOCE) in physiological and pathological thrombopoiesis. (**A**) Bone marrow, contained in spongy bones, is a tridimensional network of branching sinusoids surrounding islets of hematopoietic cells. Within this environment hematopoietic stem cells (HSCs) undergo self-renewal as well as differentiation into committed lineages in order to support the physiological homeostasis of all blood cells. Megakaryopoiesis takes place under thrombopoietin stimulation, which promotes HSC commitment and differentiation toward megakaryocytes (MKs). In MKs with replete endoplasmic reticulum (ER), Stromal Interaction Molecule (STIM) is localized in an inactive configuration in the ER membrane. Depletion of Ca^2+^ stores triggers Ca^2+^ release from the ER through inositol-trisphosphate receptors (IP3R) and consequent Ca^2+^ dissociation form STIM, which oligomerize and translocate next to the plasma membrane. Then, STIM binding to Orai and Transient Receptor Potential Canonical (TRPC) results in opening of these channels and extracellular Ca^2+^ entry. The increased cytosolic Ca^2+^ concentration ([Ca^2+^]_i_) in turn regulates cell proliferation, differentiation, migration and final platelet production; (**B**) An abnormal increase in [Ca^2+^]_i_ due to altered control of SOCE dynamics may result in pathological phenotypes such as higher proliferation and platelet production. Red arrow, increased with respect to physiological conditions.

Both PMF end ET are caused in ~10% of cases by mutations of *MPL* gene, leading to a constitutive activation of c-Mpl, the TPO receptor, and in other ~60% by mutations in *JAK2* gene, resulting in the constitutive activation of its signaling pathway which is normally activated downstream of c-Mpl upon TPO stimulation [188]. Recent findings shed new light in the genetic origin of MPNs by the description of additional somatic mutations in *CALR*, the gene encoding for the ER chaperone calreticulin, which was found in ~25% of patients with *MPL* or *JAK2* unmutated disease [189,190]. As for *MPL* and *JAK2* gene mutations, megakaryocytes expressing the mutated calreticulin present constitutive stimulation of c-Mpl downstream signaling, due to an unexpected activating interaction between the receptor and the mutated protein [191,192]. Calreticulin is a multifunctional protein that normally participates in ER Ca^2+^ storage and buffering [193]. All the mutations that have been described affect a region of the gene that encodes for the C-terminal peptide, with the resulting mutant proteins sharing a novel amino acid sequence containing positively charged amino acids, whereas the non-mutant protein is largely negatively charged [194]. In particular, a 52-base pair (bp) deletion (type-1) and a 5-bp insertion (type-2 mutation) are the most frequent variants. The discovery of these mutations triggered research at investigating the changes, if any, in Ca^2+^ homeostasis in megakaryocytes harboring the different mutations. To this regard, we demonstrated that the type-1 mutation enhanced both ER-dependent intracellular Ca^2+^ release and SOCE with respect to healthy controls, and JAK2 or type-2 mutated megakaryocytes [194]. Whether the higher SOCE magnitude could be due to the higher ER Ca^2+^ mobilization or to the end tail modifications of calreticulin mutants still remains to be elucidated. The higher intraluminal Ca^2+^ discharge is in line with the notion that, in type-1 mutation, the negatively charged amminoacids responsible for Ca^2+^ binding at the C-terminal are almost entirely replaced by either positively charged or neutral residues, while type-2 mutation does not result in the loss of stretch II and III [194]. Intriguingly, several studies reported that overexpression of calreticulin attenuated SOCE in various cell types and that this effect was likely to be mediated by the negatively charged carboxyl-terminal domain [195,196,197], thus suggesting that type-1 mutation could impair somehow SOCE machinery. Type-1 mutations are mainly associated with a significantly higher risk of myelofibrotic transformation, while type-2 mutations are preferentially associated with an indolent clinical course [194]. Based on the current knowledge about the role of SOCE in megakaryocyte physiology, we can hypothesize that high Ca^2+^ entry may have a major impact in promoting cell proliferation and migration, thus resulting in increased platelet release, justifying the augmented platelet count observed in these patients (Figure 1). Further, it has been recently described that, aside from their ability to form platelets, megakaryocytes participate to bone marrow homeostasis by releasing growth factors, such as TGF-β1, that influence ECM components deposition by both stromal cells and megakaryocytes themselves [141]. However, to which extent SOCE and mutated calreticulin may contribute to TGF-β1 and/or matrix deposition by diseased megakaryocytes remains to be clarified.

## 6. Conclusions

After 30 years form the first description of capacitative Ca^2+^ entry, many researches have been devoted to identify molecular and mechanistic process related to SOCE. In this review, we have summarized the whole machinery of SOCE, which involve STIMs, Orais and TRPCs in an elegant choreography that connects intracellular Ca^2+^ release to plasma membrane channels promoting extracellular Ca^2+^ entry. In megakaryocytes SOCE regulates fundamental cellular functions, such as proliferation, migration and sensing of extracellular environment. However, little is still known about the specific molecular and biochemical signals that are targeted by Ca^2+^ in order to drive thrombopoiesis. Understanding in details the exact mechanisms by which SOCE control megakaryocyte functions would be instrumental for the identification of novel pathways that may be involved in the pathogenesis of megakaryocyte-related diseases.

## Abbreviations

ADP	Adenosine diphosphate
ATP	Adenosine triphosphate
Ca^2+^	Calcium
[Ca^2+^]_i_	Cytoplasmic calcium concentration
CALR	Calreticulin
CC	Coiled-coil
CD	Cluster of differentiation
CDI	Calcium-dependent inactivation
cEF	Canonical EF-hand domain
CIRB	Calmodulin/inositol 1,4,5-trisphosphate receptor binding region
CRAC	Calcium-release activated calcium channel
Cs^+^	Cesium
c-Mpl	Thrombopoietin receptor
DAG	Diacylglycerol
DMS	Demarcation membrane system
dOrai	*Drosophila* Orai
ECM	Extracellular matrix
*E*_rev_	Reversal potential
ET	Essential thrombocythemia
GPCR	G-protein coupled receptor
hEF	Hidden non-canonical EF-hand domain
HSC	Hematopoietic stem cells
HSG	Human salivary gland
I_CRAC_	Calcium release-activated calcium current
IP3	Inositol 1,4,5-trisphosphate
IP3R	Inositol 1,4,5-trisphosphate receptor
I_SOC_	Store-operated activated calcium current
JAK2	Janus kinase 2
K^+^	Potassium
MPN	Phildelphia-negative myeloproliferative neoplasm
Na^+^	Sodium
NCX	Sodium/calcium exchanger
NF-κB	Nuclear factor κ-light-chain-enhancer of activated B cells
NMDAR	Ionotropic *N*-methyl-d-aspartate receptor
PDGF	Platelet derived growth factor
PF4	Platelet factor 4
PIP2	Phosphatidylinositol 4,5-bisphosphate
PKC	Protein kinase C
PKG	Protein kinase G
PLC	Phospholipase C
PMCA	Plasma membrane calcium-ATPase
PMF	Primary myelofibrosis
RNA	Ribonucleic acid
ROC	Receptor-operated channel
RyR	Ryanodine receptors
SAM	Sterile-α motif
SDF-1α	Stromal derived factor-1α
SERCA	Sarco/endoplasmic reticulum calcium-ATPase
SGK1	Serum- and glucocorticoid-inducible kinase 1
SMOC	Second messenger-operated channel
SOC	Store-operated channel
SOCE	Store-operated calcium entry
SR/ER	Sarco/endoplasmic reticulum
STIM	Stromal interaction molecule
TGF-β1	Transforming growth factor-β1
TKR	Tyrosine kinase receptor
TM	Transmembrane
TPO	Thrombopoietin
TRPA-1	Transient receptor potential ankyrin-1
TRPC	Transient receptor potential canonical
TRPV	Transient receptor potential vanilloid
VEGF	Vascular endothelial growth factor
VOC	Voltage-operated channel
vWF	Von Willebrand Factor
